# Pulse Pressure Variation Can Predict the Hemodynamic Response to Pneumoperitoneum in Dogs: A Retrospective Study

**DOI:** 10.3390/vetsci6010017

**Published:** 2019-02-20

**Authors:** Caterina Di Bella, Luca Lacitignola, Laura Fracassi, Despoina Skouropoulou, Antonio Crovace, Francesco Staffieri

**Affiliations:** 1 PhD Course in Tissues and Organs Transplantation and Cellular Therapies, Department of Emergency and Organ Transplantation, University of Bari, 70010 Bari, Italy; l.fracassi123@gmail.com (L.F.); semeli.amenta@gmail.com (D.S.); 2 Department of Emergency and Organ Transplantation, Section of Veterinary Clinics and Animal Production; University of Bari, 70010 Bari, Italy; luca.lacitignola@uniba.it (L.L.); antonio.crovace@uniba.it (A.C.); francesco.staffieri@uniba.it (F.S.)

**Keywords:** hemodynamic, pneumoperitoneum, dogs, fluid, anesthesia

## Abstract

Pneumoperitoneum may induce important hemodynamic alterations in healthy subjects. Pulse pressure variation (PPV) is a hemodynamic parameter able to discriminate preload dependent subjects. Anesthesia records of dogs undergoing laparoscopy were retrospectively evaluated. The anesthetic protocol included acepromazine, methadone, propofol and isoflurane administered with oxygen under mechanical ventilation. The hemodynamic parameters were considered five minutes before (BASE) and ten minutes after (P10) the pneumoperitoneum. Based on the cardiac index (CI) variation, at P10, dogs were classified as sensitive (S group, CI ≤ 15%) and non-sensitive (NO-S group). Data were analyzed with the ANOVA test and the ROC curve (*p* < 0.05). Fifty-five percent of dogs (S) had a reduction of CI ≥ 15% at P10 (2.97 ± 1.4 L/min/m^2^) compared to BASE (4.32 ± 1.62 L/min/m^2^) and at P10 in the NO-S group (4.51 ± 1.41 L/min/m^2^). PPV at BASE was significantly higher in the S group (22.4% ± 6.1%) compared to the NO-S group (10.9% ± 3.3%). The ROC curve showed a threshold of PPV > 16% to distinguish the S and NO-S groups. PPV may be a valid predictor of the hemodynamic response to pneumoperitoneum in dogs. A PPV > 16% can identify patients that may require fluid administration before the creation of pneumoperitoneum.

## 1. Introduction

During intermittent positive pressure ventilation (IPPV), alterations in intra-thoracic pressure are transmitted to the heart, inducing cyclic changes in the loading conditions of the right and left ventricles (heart lung interaction) [1]. Specifically, positive pressure inspiration decreases preload and increases afterload of the right ventricle (RV), leading to a temporary decrease in stroke volume [2]. Accordingly, the left ventricular (LV) preload reduction induces a decrease in LV filling and a subsequent reduction in stroke volume (SV), which will be evident during expiration due to the lag phase of two to three heart beats because of the pulmonary transit time [2]. The entity of the variation of SV during IPPV is related to the volemic status of the cardiovascular system, being more important in subjects on the step portion of the Frank–Starling curve (preload dependent) [3]. The arterial pulse pressure (the difference between the systolic and diastolic pressures) is directly proportional to SV and its variation in relation to the respiratory cycle (pulse pressure variation, PPV) has been proven to be predictive of the response to the administration of a bolus of fluids (fluid challenge) [3,4]. In human patients, a PPV value equal to or above 13% has been proven to indicate preload dependency; in particular, patients respond with an increase in SV after fluid infusion [5,6]. Recently, evaluation of PPV has gained popularity, including in veterinary medicine to guide volume expansion [7]. In dogs, PPV was shown to be accurate for predicting fluid responsiveness in a model of hemorrhagic shock [8] and in mechanically ventilated isoflurane-anesthetized dogs pre-medicated with acepromazine [9]. These studies showed that changes in PPV and hypovolemic conditions occurred much earlier than changes in classical parameters such as arterial blood pressure, heart rate (HR), or central venous pressure [7,8]. In a clinical study in dogs, Fantoni et al. [10] showed that PPV can predict fluid responsiveness in dogs undergoing orthopedic surgery, and they found a cut-off value of 15% distinguishing responders from non-responders. Drozdzynka et al. [7] used a PPV of 13% as a cut-off for the intraoperative administration of a fluid bolus as part of a goal-directed protocol in dogs undergoing abdominal surgery, showing that a PPV ≥ 13% reliably predicted the cardiovascular response to fluid loading in 82.8% of canine cases undergoing abdominal surgery, in agreement with findings from human studies [2].

The increase of intra-abdominal pressure (IAP) during CO_2_ pneumoperitoneum (PP) causes several cardiovascular perturbations headed by the reduction of the venous return [11]. From the right ventricle perspective, the rapid decrease of the end diastolic volume, determined by PP, will cause a reduction of the RV stroke volume and of the cardiac output (CO), which will be more important in preload dependent subjects [12,13,14]. The mechanism for the decrease of cardiac output is multifactorial and is related to an increased IAP, which results in caval compression, pooling of blood in the periphery, and an increase in venous resistance [11,15].

Based on this background, the rationale of our study was to evaluate whether the PPV value, before the induction of PP, could be a predictor of the hemodynamic response to laparoscopy in dogs. Our hypothesis is that in preload dependent subjects, higher values of PPV, before the creation of PP, can be predictive of a significant hemodynamic derangement during the procedure, and thus may identify dogs needing cardiovascular stabilization before starting the procedure. To test this hypothesis, the PPV and the hemodynamic values of a series of dogs undergoing laparoscopic ovariectomies were retrospectively evaluated.

## 2. Materials and Methods 

The study was approved by the Ethical Committee for Clinical Study in Animal Patients of the Department of Emergency and Organ Transplantation of the University of Bari (n. 03/2016). In this retrospective study, the anesthesia records of 32 cases of dogs undergoing elective laparoscopic ovariectomies were recruited between January and May 2018 at the Department of Emergency and Organ Transplantation, Section of Veterinary Clinics and Animal Production, “Aldo Moro” University of Bari, Bari, Italy. Dogs affected by systemic or cardiovascular disease were excluded along with cases in which the collection of data was incomplete. 

### 2.1. Anesthetic Protocol

All subjects were premedicated following the standard protocol used for this procedure in healthy dogs (ASA1) at our institution, which included premedication with 10 μg/kg of acepromazine intramuscularly (IM, Prequillan; Fatro, Italy; 10 mg/mL) followed after 15 min by 0.3 mg/kg IM of methadone (Semfortan; Dechra, Italy; 10 mg/mL). The cephalic vein was cannulated for the administration of propofol (Fresenius Kabi Propofol 10 mg/mL) at 5 mg/kg IV, fluids (Ringer Lactate solution; Fresenius Kabi) at 5 mL/kg/h for the entire duration of the procedure, and other drugs, as required. General anesthesia was maintained with inhaled isoflurane in oxygen (FiO_2_ > 0.8). All dogs were mechanically ventilated in a volume-controlled mode (Servo-I; Maquet, Germany), with a tidal volume (TV) of 15 mL/kg, inspiratory to expiratory ratio of 1:2, inspiratory pause of 25% of inspiratory time, and positive end-expiratory pressure (PEEP) of 0 cmH_2_O. The respiratory rate (RR) was adjusted for the end-tidal carbon dioxide level (EtCO_2_), which was maintained between 40 and 55 mmHg. The following respiratory (DatexOhmeda S/5 Anesthesia Monitor, Ohmeda, Soma Technology, Bloomfield, CT, USA) and hemodynamic (PRAM, Most Care^®^, Vytech, Padova, Italy) parameters, manually collected every five minutes during the procedure, were considered for the study: peripheral capillary oxygen hemoglobin saturation (SpO2, %); TV (mL/kg); EtCO_2_, (mmHg); peak and plateau airway pressures (Ppeak and Pplat, cmH_2_O); RR (breaths/minute); static compliance of the respiratory system (Crs, mL/cmH_2_O/kg); HR (beats/minute); systolic, mean and diastolic arterial pressures (SAP, MAP and DAP respectively; mmHg); SV (mL); CO (L/min); systemic vascular resistances (SVR, dynes*seconds/cm^5^); and PPV(%). The end-tidal concentration of isoflurane (EtIso, %) and the temperature (T, °C) were also considered. The hemodynamic parameters were collected with a monitoring system based on the pressure recording analytical method technology (PRAM). This uncalibrated pulse contour technique estimates SV and other hemodynamic parameters from the analysis of the arterial pulse waveform, and has been recently validated in dogs [16]. In all cases of the study, PP was created via a Veress needle with a CO_2_ insufflator (Endoflator; Karl-Storz, Tuttlingen, Germany) at an IAP of 10–11 mmHg. 

### 2.2. Study Protocol

For the purpose of the study, the physiological data registered five minutes before (BASE) and ten minutes after (P10) the induction PP were considered; in particular, the following cardiovascular parameters were recorded: HR, MAP, SV, CO, SVR, and PPV. These data were automatically stored every 3 s by the hemodynamic monitor, which allowed further offline analysis. 

The cardiac index (CI, L/minute/m^2^) was calculated using the formula

CI = CO/BSA(1)

where BSA represents the body surface area (m^2^).

PPV was automatically calculated by the software of the monitor using the following formula:

PPV(%) = 100*(PP_max_ − PP_min_)/[(PP_max_ − PP_min_)/2] (2)

where PP represents the difference between the systolic and diastolic pressure, and PP_max_ and PP_min_ indicate the maximum and minimum value of PP in a single respiratory cycle, respectively. 

### 2.3. Statistical Analysis

All data were analyzed by MedCalc Software 9.2 (MedCalc, Mariakerke, Belgium). The Shapiro–Wilk test was used to evaluate the normal distribution of the data collected at BASE and P10. All parameters were reported as mean ± standard deviation (SD). The differences between BASE and P10 were tested for the entire population with the paired samples Student’s t-test. Based on the variation of the CI, following the creation of the PP, the cases were divided into two groups. Those presenting a reduction of CI equal to or greater than 15% were considered as sensitive (S). Otherwise, they were considered non-sensitive (NO-S). The comparison between the two groups (S and NO-S) at BASE and P10 was performed with the one-way ANOVA for repeated measurements. A receiver operating characteristic (ROC) curve was generated for PPV at BASE in order to discriminate S from NO-S; the area under the ROC curve was calculated with 95% confidence intervals and the optimal threshold value (the value that maximizes the sum of both sensitivity and specificity) for PPV was determined. Statistical significance was set at *p* < 0.05.

## 3. Results

Twelve cases were discarded because either they referred to subjects suffering from systemic or cardiovascular diseases (four cases), or the data collected were not complete (eight cases). Accordingly, 20 cases formed the population of the study with a mean body weight and age of 16.4 ± 8.1 kg and 3.3 ± 1.2 years, respectively. The breeds included were beagle (*n* = 4), cocker spaniel (*n* = 3), bull terrier (*n* = 2) deutscher boxer (*n* = 2), and mixed breed (*n* = 9) (Table 1).

The average values of the cardiovascular and respiratory parameters registered before and after PP are reported in Table 2.

After the PP, CI, PPV, and Crs decreased, while MAP, Ppeak and Pplat increased significantly. All other physiological parameters did not exhibit significant differences between the two phases of the study. Eleven (55%) of the 20 subjects were assigned to the S group because they showed a decrease of the CI ≥ 15%; the remaining nine dogs (45%) were ascribed to the NO-S group. Specifically, the mean and SD of the variation of CI after the induction of PP was –32.3% ± 15.2% in the S group, while in the NO-S group it was at 7.18% ± 13.66%. The body weight and age of the two groups were similar (S: 14.6 ± 7.2 kg and 3.2 ± 1.2 years; NO-S: 18.3 ± 9.1 kg and 3.5 ± 1.2 years). The cardiovascular parameters and the EtCO_2_ recorded in the two groups at the different times of the study are reported in Table 3.

The cardiac index and SVR were similar between the two groups at BASE, but at P10 the CI was lower in group S compared to the NO-S group, and the SVR was lower in the NO-S group compared to the S group (Table 3).

Pulse pressure variation at BASE was greater in the S group compared to the NO-S group (Table 3), while it was similar between the two groups at P10.

**Figure 1:**
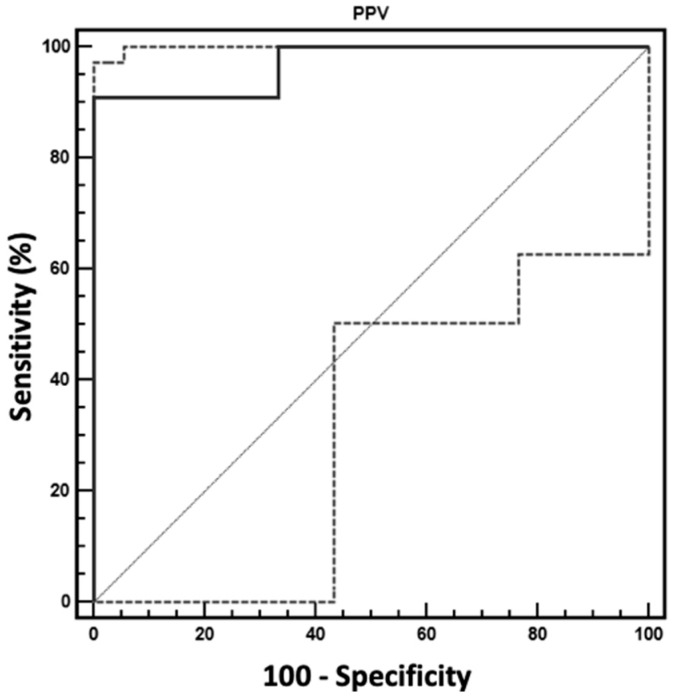
Graphical representation of the ROC curve to test the ability of pulse pressure variation (PPV) to discriminate between sensitive (S group = decrease in CI ≥ 15% after PP) and non-sensitive (NO-S) subjects after the creation of the pneumoperitoneum in dogs undergoing laparoscopic surgery.

Heart rate and MAP were similar between the two groups at the two evaluation times. The mean values of PPV and CI at P10 in the S group were lower than the corresponding values at BASE. The PPV showed a significant area under the ROC curve (0.970 ± 0.039; *p* = 0.0001). The best cutoff of PPV was 16%, with a sensitivity of 90.91% and a specificity of 100% (confidence interval 0.780–0.987) to distinguish between S and NO-S groups (Figure 1).

## 4. Discussion

The results of this study demonstrate that PPV could be a valuable parameter to predict the hemodynamic response to PP in dogs. In particular, a PPV value higher than 16% may predict a significant reduction of CO in response to the creation of the pneumoperitoneum; thus, it identify subjects who may benefit from fluids administration before CO_2_ insufflation.

The hemodynamic perturbations observed during laparoscopy occured mainly at the beginning of the procedure and resulted from the combined effects of pneumoperitoneum, patient position, anesthesia, and hypercapnia from the absorbed CO_2_. The typical disturbances are characterized by decreases in CO proportional to the increase of the IAP, increased arterial pressure, and elevation of systemic and pulmonary vascular resistances; heart rate remains unchanged or increases slightly [17,18,19]. Cardiac output has also been reported to be increased or unchanged during pneumoperitoneum in human patients and dogs; these discrepancies might be related to differences in rates of CO_2_ insufflation, IAP, time intervals between insufflation, and differences in data collection and anesthetic technique and drugs [20,21,22]. The absolute or relative intravascular blood volume is an important factor determining the hemodynamic response to PP, and preload-dependent patients usually experience the most severe hemodynamic side effects during laparoscopy [23]. In these patients, the reduction of venous return and CO can be attenuated by increasing circulating volume before the PP is produced [11,24]. The results of this retrospective study showed that 55% (S group) of healthy dogs undergoing laparoscopy might experience an important derangement of the CO 10 min after CO_2_ insufflation. Interestingly, these dogs did not show any significant hemodynamic differences (CI, MAP, SVR and HR) compared to dogs in the NO-S group before the PP production, except that they had a higher value of PPV. The PPV during positive pressure ventilation is a hemodynamic index able to discriminate preload-dependent subjects that need fluid administration [3]. Thus, we can suppose that the hearts of dogs in the S group were in a compensated preload-dependent condition before PP, and thereafter, the decrease in venous return induced by the PP caused an additional cardiovascular derangement determining an important reduction of the CI. Systemic vascular resistances were similar between the two groups at BASE, but during the PP they were higher in the S group, confirming that these subjects were attempting to compensate for the cardiovascular perturbation created by the PP. All cases included in this study were elective ovariectomy and were healthy. The fact that dogs of the S group resulted as being preload dependent based on the PPV analysis does not necessarily mean they were hypovolemic. Indeed, a preload-dependent condition can also be related to the response of the subjects to the effects of drugs (e.g., acepromazine, isoflurane) or the anesthetic technique (e.g., mechanical ventilation).

The analysis of the ROC curve indicated that a PPV value equal to or higher than 16% before the CO_2_ insufflation is predictive of an important hemodynamic derangement during the PP, with a high sensibility and specificity. Thus, this threshold of PPV could be useful in clinical cases not only to identify dogs at risk of cardiovascular side effects to PP, but also to guide fluid therapy. Indeed, in these subjects fluids should be administered in order to reduce the PPV to values lower than 16%. Further prospective studies are required to confirm whether this approach could be valid in clinical cases. The literature indicates that in dogs not subjected to laparoscopy, the PPV cut-off value to discriminate “preload dependent” subjects is lower (11%–15%) [7,9,10] than the value found in this study. We can suppose that the difference is related to the impact of PP, which added an additional factor influencing the hemodynamic status compared to dogs not subjected to laparoscopy.

This retrospective study identified a possible monitoring value of PPV in predicting important negative hemodynamic effects of PP. Future prospective studies need to confirm that in these cases (PPV > 16%) fluid administration will prevent the hemodynamic derangement caused by PP. Another limitation of the study is that the dogs evaluated were healthy; thus, it is possible that there are different cut-off values for hemodynamically unstable cases. Moreover, variability on the cardiovascular response can be expected based on breed, age, and sex, but the limited number of cases did not allow this study to consider those factors.

## 5. Conclusions

Monitoring of PPV is very valuable during laparoscopy in dogs, since it can predict adverse cardiovascular reaction to PP. Moreover, PPV could be used to optimize the fluid therapy of dogs before the production of PP. Values of PPV higher than 16% before the insufflation of CO_2_ are predictive of cardiovascular side effects to PP, and thus, should suggest prior fluid administration.

## Figures and Tables

**Table 1:** Breed, age, heart rate (HR), mean arterial pressure (MAP), cardiac index (CI), pulse pressure variation (PPV), and respiratory system compliance (Crs) of the cases included in the study, immediately before (BASE) and 10 min after the pneumoperitoneum (P10). Dogs that showed a reduction of CI ≥ 15% at P10 compared to BASE were classified as sensitive (S), the rest of the dogs were classified as non-sensitive (NO-S).

Dog	Phase	Breed	Age (Years)	HR (beats/min)	MAP (mmHg)	CI (L/min/m^2^)	PPV (%)	Crs (mL/cmH_2_O/kg)	Group
1	BASE	cocker spaniel	2	87	98	2.9	21	0.9	S
	P10			86	117	1.1	11	0.8	
2	BASE	mixed breed	3	72	79	5.9	32	1.0	S
	P10			96	76	2.6	12	0.8	
3	BASE	mixed breed	3	75	79	2.2	18	1.6	S
	P10			84	128	1.4	7	0.9	
4	BASE	bull terrier	4	41	76	6.3	22	1.6	S
	P10			85	121	4.7	21	0.9	
5	BASE	beagle	4	108	64	6.5	13.6	2.1	S
	P10			109	89	4.5	18	0.6	
6	BASE	beagle	2	88	86	5.6	19	1	S
	P10			102	54	4.7	13	0.9	
7	BASE	mixed breed	4	116	76	2.7	20	1.1	S
	P10			131	59	1.6	7	1.1	
8	BASE	boxer	5	128	88	3.4	26	1.1	S
	P10			120	100	2.6	16	0.6	
9	BASE	cocker spaniel	2	72	75	3.6	33	1.8	S
	P10			83	82	2.3	12	0.9	
10	BASE	mixed breed	1	99	54	2.7	24	1.4	S
	P10			78	95	2.1	7.8	0.8	
11	BASE	beagle	4	71	81	5.3	18	1.9	S
	P10			71	91	4.6	12	0.7	
12	BASE	mixed breed	3	107	81	4.6	6.3	3.1	NS
	P10			101	87	4.4	5	1.1	
13	BASE	cocker spaniel	5	56	70	7.5	11	2.3	NS
	P10			80	99	7.6	10	1.3	
14	BASE	mixed breed	2	102	83	3.7	7.3	0.9	NS
	P10			73	74	4.1	10	0.5	
15	BASE	mixed breed	2	60	76	3.5	8.8	1.4	NS
	P10			91	96	4.6	7	1.2	
16	BASE	beagle	5	84	80	3.2	14	1.9	NS
	P10			90	101	3.7	9	1.2	
17	BASE	bull terrier	4	67	68	4.7	14	1.3	NS
	P10			89	100	5.7	9	1	
18	BASE	boxer	6	97	70	3.6	9	1.4	NS
	P10			89	95	3.3	14	1.4	
19	BASE	mixed breed	3	76	70	4.2	11	1.6	NS
	P10			102	76	4.4	9	1.3	
20	BASE	mixed breed	2	91	83	2.8	16	1.7	NS
	P10			76	96	2.6	7	1.1	

**Table 2:** Mean ± SD of the cardiovascular and respiratory parameters evaluated 5 min before (BASE) and 10 min after (P10) the pneumoperitoneum (PP) in twenty mechanically ventilated isoflurane-anesthetized dogs. * *p* < 0.05 between the two evaluation times of the study.

Parameter	BASE	P10	*p* Value
HR (beats/min)	84.8 ± 21.6	91.8 ± 15.4	0.111
MV (L/min/kg)	0.17 ± 0.03	0.19 ± 0.04	0.127
MAP (mmHg)	76.8 ± 9.4	91.8 ± 18.5	0.003 *
CI (L/min/m^2^)	4.28 ± 1.4	3.67 ± 1.6	0.019 *
SVR (dyn*sec/cm^5^)	3117 ± 1485	3003 ± 1341	0.775
PPV (%)	17.2 ± 7.6	11.1 ± 4.0	0.001*
EtCO2 (mmHg)	45.9 ± 4.9	52.6 ± 7.4	0.067
SpO2 (%)	98.2 ± 1.1	97.6 ± 1.4	0.765
Crs (mL/cmH_2_O/kg)	1.6 ± 0.6	0.9 ± 0.2	0.001 *
Ppeak (cmH_2_O)	8.9 ± 1.8	12.3 ± 2.9	0.001 *
Pplat (cmH_2_O)	8.7 ± 1.8	11.6 ± 2.3	0.001 *

**Table 3:** Mean ± SD of the cardiovascular parameters evaluated in mechanically ventilated isoflurane-anesthetized dogs, 5 min before (BASE) and 10 min after (P10) the pneumoperitoneum. Based on the variation of CI after the PP, dogs were divided into the groups S (CI decreased ≥ 15%) and NO-S. The *p* value related to the comparison of the two groups at the same time is also reported in table. * *p* < 0.05 between the two groups. ^#^ *p* < 0.05 compared to the corresponding basal value.

Parameter	Phase	S Group	NO-S Group	*p* Value
HR (beats/min)	BASE	86.4 ± 25.1	81.1 ± 20.1	0.601
	P10	96.1 ± 26.8	94.1 ± 12.5	0.822
MAP (mmHg)	BASE	77.9 ± 11.6	74.6 ± 6.8	0.471
	P10	89.1 ± 27.1	92.1 ± 7.4	0.742
CI (L/m^2^)	BASE	4.32 ± 1.62	4.22 ± 1.38	0.88
	P10	2.97 ± 1.4 ^#^	4.51 ± 1.41	0.02 *
SVR (dyn*sec/cm^5^)	BASE	3081 ± 1007	3160 ± 1991	0.902
	P10	3573 ± 1245	2305 ±1138	0.032 *
EtCO_2_	BASE	46.2 ± 5.24	43.2 ± 7.2	0.602
	P10	50.1 ± 7.32	48.2 ±5.6	0.732
PPV (%)	BASE	22.4 ± 6.1	10.9 ± 3.3	0.000 *
	P10	12.6 ± 4.3 ^#^	9.1 ± 2.5	0.05

## References

[1] Cheifetz I.M. (2014). Cardiorespiratory interactions: The relationship between mechanical ventilation and hemodynamics. Respir. Care.

[2] Michard F. (2005). Changes in arterial pressure during mechanical ventilation. Anesthesiology.

[3] Cannesson M. (2010). Arterial pressure variation and goal-directed fluid therapy. J. Cardiothorac. Vasc. Anesth..

[4] Yang X., Du B. (2014). Does pulse pressure variation predict fluid responsiveness in critically ill patients? A systematic review and meta-analysis. Crit. Care.

[5] Perel A., Pizov R., Cotev S. (2014). Respiratory variations in the arterial pressure during mechanical ventilation reflect volume status and fluid responsiveness. Intensive Care Med..

[6] Marik P.E., Cavallazzi R., Vasu T., Hirani A. (2009). Dynamic changes in arterial waveform derived variables and fluid responsiveness in mechanically ventilated patients: A systematic review of the literature. Crit. Care Med..

[7] Drozdzynska M.J., Chang Y.M., Stanzani G., Pelligand L. (2018). Evaluation of the dynamic predictors of fluid responsiveness in dogs receiving goal-directed fluid therapy. Vet. Anaesth. Analg..

[8] Berkenstadt H., Friedman Z., Preisman S., Keidan I., Livingstone D., Perel A. (2005). Pulse pressure and stroke volume variations during severe haemorrhage in ventilated dogs. Br. J. Anaesth..

[9] Sano H., Seo J., Wightman P., Cave N.J., Gieseg M.A., Johnson C.B., Chambers P. (2018). Evaluation of pulse pressure variation and pleth variability index to predict fluid responsiveness in mechanically ventilated isoflurane-anesthetized dogs. J. Vet. Emerg. Crit. Care.

[10] Fantoni D.T., Ida K.K., Gimenes A.M., Mantovani M.M., Castro J.R., Patricio G.C.F., Ambrosio A.M., Otsuki D.A. (2017). Pulse pressure variation as a guide for volume expansion in dogs undergoing orthopedic surgery. Vet. Anaesth. Analg..

[11] Kashtan J., Green J.F., Parsons E.Q., Holcroft J.W. (1981). Hemodynamic effect of increased abdominal pressure. J. Surg. Res..

[12] Zuckerman R.S., Heneghan S. (2002). The duration of hemodynamic depression during laparoscopic cholecystectomy. Surg. Endosc..

[13] Liu F., Zhu S., Ji Q., Li W., Liu J. (2015). The impact of intra-abdominal pressure on the stroke volume variation and plethysmographic variability index in patients undergoing laparoscopic cholecystectomy. Biosci. Trends.

[14] Branche P.E., Duperret S.L., Sagnard P.E., Boulez J.L., Petit P.L., Viale J.P. (1998). Left ventricular loading modifications induced by pneumoperitoneum: A time course echocardiographic study. Anesth. Analg..

[15] Takata M., Wise R.A., Robotham J.L. (1990). Effects of abdominal pressure on venous return: Abdominal vascular zone conditions. J. Appl. Physiol..

[16] Briganti A., Evangelista F., Centonze P., Rizzo A., Bentivegna F., Crovace A., Staffieri F. (2018). A preliminary study evaluating cardiac output measurement using Pressure Recording Analytical Method (PRAM) in anaesthetized dogs. BMC Vet. Res..

[17] Struthers A.D., Cuschieri A. (1998). Cardiovascular consequences of laparoscopic surgery. Lancet.

[18] Koivusalo A.M., Lindgren L. (2000). Effects of carbon dioxide pneumoperitoneum for laparoscopic cholecystectomy. Acta Anaesthesiol. Scand..

[19] Atkinson T.M., Giraud G.D., Togioka B.M., Jones D.B., Cigarroa J.E. (2017). Cardiovascular and Ventilatory Consequences of Laparoscopic Surgery. Circulation.

[20] Ivankovich A.D., Miletich D.J., Albrecht R.F., Heyman H.J., Bonnet R.F. (1975). Cardiovascular effects of intraperitoneal insufflation with carbon dioxide and nitrous oxide in the dog. Anesthesiology.

[21] Duke T., Steinacher S.L., Remedios A.M. (1996). Cardiopulmonary effects of using carbon dioxide for laparoscopic surgery in dogs. Vet. Surg..

[22] Dexter S.P., Vucevic M., Gibson J., McMahon M.J. (1999). Hemodynamic consequences of high- and low-pressure capnoperitoneum during laparoscopic cholecystectomy. Surg. Endosc..

[23] Safran D., Sgambati S., Orlando R. (1993). Laparoscopy in high-risk cardiac patients. Surg. Gynecol. Obstet..

[24] Ho H.S., Saunders C.J., Corso F.A., Wolfe B.M. (1993). The effects of CO_2_ pneumoperitoneum on hemodynamics in hemorrhaged animals. Surgery.

